# Orthotopic Porcine Liver and Kidney Xenotransplantation as a Step Toward Multi-Organ Replacement

**DOI:** 10.7150/ijbs.139541

**Published:** 2026-08-24

**Authors:** Hwijin Kim, Jongwon Byun, Sun-Uk Kim, Taeho Kwon, Kyungjun Uh

**Affiliations:** 1 Futuristic Animal Resource and Research Center, Korea Research Institute of Bioscience and Biotechnology (KRIBB), Cheongju, Chungbuk 28116, Republic of Korea.; 2 Advanced Bioconvergence Department, KRIBB School, Korea National University of Science and Technology (UST), Daejeon 34113, Republic of Korea.; 3 Functional Genomics Department, KRIBB School, Korea National University of Science and Technology (UST), Daejeon 34113, Republic of Korea.

In the study discussed here, Liao *et al*. reported orthotopic multi-organ xenotransplantation of a porcine whole liver and bilateral kidneys from a pig carrying six genetic modifications into a human decedent model 1. This work addresses a central question in xenotransplantation, namely whether multiple porcine organs can maintain functional and structural integrity after simultaneous orthotopic transplantation in a human physiological environment.

The shortage of organs available for transplantation remains one of the most important challenges facing modern medicine. Xeno-transplantation has therefore emerged as a potential strategy to expand the donor pool, and pigs are considered promising alternative donors because of their anatomical and physiological compatibility with humans and the possibility of reducing immune barriers through genetic modification 2. However, orthotopic multi-organ xenotransplantation is fundamentally different from single-organ xeno-transplantation. Combined liver and kidney trans-plantation requires coordinated restoration of hepatic metabolic and synthetic function together with renal excretory homeostasis 3. In xeno-transplantation, this complexity is further amplified because immune responses, hemodynamics, coagulation, and metabolic activity interact across species barriers.

Recent human decedent studies, including gene-modified pig to human liver xenotransplantation and the first pig to human lung xenotransplantation, have accelerated clinical interest in xenotransplantation 4-7. Against this background, Liao *et al*. extended the field from single-organ studies toward simultaneous orthotopic multi-organ replacement in two important ways 1. First, the authors performed orthotopic multi-organ xenotransplantation of a porcine whole liver and bilateral kidneys in a human decedent model. Unlike previous xenotransplantation studies that mainly focused on auxiliary or single-organ transplantation, this study examined the replacement of the native liver and kidneys with multiple porcine grafts in an orthotopic setting 4, 6, 8.

The donor pig carried knockouts of *GGTA1*, *CMAH*, and *B4GALNT2* to reduce major xenoantigens, together with insertion of *hCD46*, *hCD55*, and *hTHBD* to regulate complement activation and coagulation. Second, by integrating single-cell RNA sequencing (scRNA-seq), CellChat analysis, liquid chroma-tography-tandem mass spectrometry (LC-MS/MS) metabolomics, proteomics, histological assessment, and clinical monitoring, the authors evaluated early graft function, immune responses, and systemic metabolic responses in a multidimensional manner. Thus, the significance of this study lies not only in surgical feasibility but also in providing a framework for analyzing how multiple porcine organs function in a human physiological environment.

Functionally, the porcine liver and kidney xenografts demonstrated measurable early activity. The liver produced bile, while circulating albumin levels were maintained, although the latter alone does not establish the extent of porcine hepatic albumin synthesis. Alanine aminotransferase remained within the normal range, whereas aspartate aminotransferase transiently increased and then declined. Renal function also improved, as reflected by decreases in serum creatinine and urea and by an increase in the estimated glomerular filtration rate (eGFR). Plasma uric acid decreased during the early observation period, although interpretation of this change is complicated by porcine hepatic uricase activity and differences in purine metabolism between species.

Another important observation was the absence of classical hyperacute rejection (HAR) during the initial observation period. Deposition of the complement activation products C3d and C5b-9 was negligible, IgM deposition was absent, and only minimal IgG deposition was observed. These findings suggest that multiplex genome editing combined with perioperative immunosuppression was sufficient to prevent classical HAR during the early observation period, although whether this translates into long-term immunological stability remains unknown. Nevertheless, the appearance of microthrombi after 36 hours indicates that coagulation incompatibility was not completely resolved.

In terms of immune analysis, a notable finding was the time-dependent expansion and graft infiltration of S100A12⁺ neutrophils in both peripheral blood and transplanted tissues. CellChat and ligand-receptor analyses inferred interactions between this population and other immune cells, with the adhesion G protein-coupled receptor E family (ADGRE) signaling axis emerging as a candidate network. However, the predicted ligand-receptor interactions and the putative role of the ADGRE-associated network were derived solely from computational analysis of single-cell transcriptomic data from a single case. These inferences were not independently validated at the molecular or functional level and should therefore be regarded as hypothesis-generating findings rather than as evidence of a defined mechanism or validated therapeutic targets. Functional studies, such as receptor blockade, selective depletion of neutrophils, or *in vitro* co-culture assays, will be needed to determine whether S100A12⁺ neutrophils and the ADGRE-associated network contribute to xenograft injury or instead reflect a broader inflammatory response during the perioperative period.

Metabolomic profiling is another notable aspect of this study. Untargeted LC-MS/MS analysis showed that post-transplant metabolic profiles remained more closely aligned with the recipient's pre-transplant baseline than with the donor pig profile. However, this similarity should not be interpreted as direct evidence of intrinsic metabolic adaptation by the porcine grafts. Because the grafts were continuously perfused by the recipient's circulation, the measured metabolic profile may partly reflect circulating host-derived metabolites rather than intrinsic metabolic reprogramming of the porcine grafts. The absence of a parallel human allotransplantation control further limits the distinction between metabolic changes specific to xenotransplantation and general responses to major surgery, injury caused by ischemia followed by reperfusion, and immunosuppression.

This study has several important limitations. Most fundamentally, all data derive from a single human decedent recipient transplanted with organs from a single porcine donor. In addition, the 106-hour observation period was meaningful for evaluating early immune responses and functional viability but was insufficient to assess chronic rejection, durable coagulation control, or long-term metabolic adaptation. The hormonal and inflammatory state of the human decedent recipient may also have influenced the findings. Because the liver and both kidneys were transplanted simultaneously, the individual contributions of each graft to systemic immune and metabolic changes could not be distinguished. Furthermore, the absence of hyperacute rejection must be interpreted in the context of negative screening for xenoreactive antibodies before transplantation, and its generalizability will require evaluation in expanded cohorts.

Nevertheless, this study provides the first evidence from a human decedent model supporting the technical feasibility of orthotopic multi-organ xenotransplantation over a short observation period and offers multidimensional immune and metabolic data that may inform future clinical translation. Future studies should establish reproducibility through expanded case series, extend the observation period, incorporate comparative allotransplantation controls, and functionally validate the biological relevance of S100A12⁺ neutrophil expansion and the inferred ADGRE-associated interactions. From a translational standpoint, the early immune and coagulation signatures observed here, particularly the expansion of S100A12⁺ neutrophils and the delayed appearance of microthrombi, may inform monitoring priorities and the selection of safety endpoints for future clinical studies of multi-organ xenotransplantation. Such studies will require prespecified functional endpoints, carefully defined recipient selection criteria, and intensive immune and coagulation monitoring. The significance of this study does not lie in demonstrating immediate clinical applicability. Rather, it shows that porcine liver and kidney grafts can provide measurable support for multiple organ functions over a short period in a human decedent model and identifies immune, coagulation, and metabolic axes that require systematic investigation before durable clinical replacement can be considered.

## Figures and Tables

**Figure 1:**
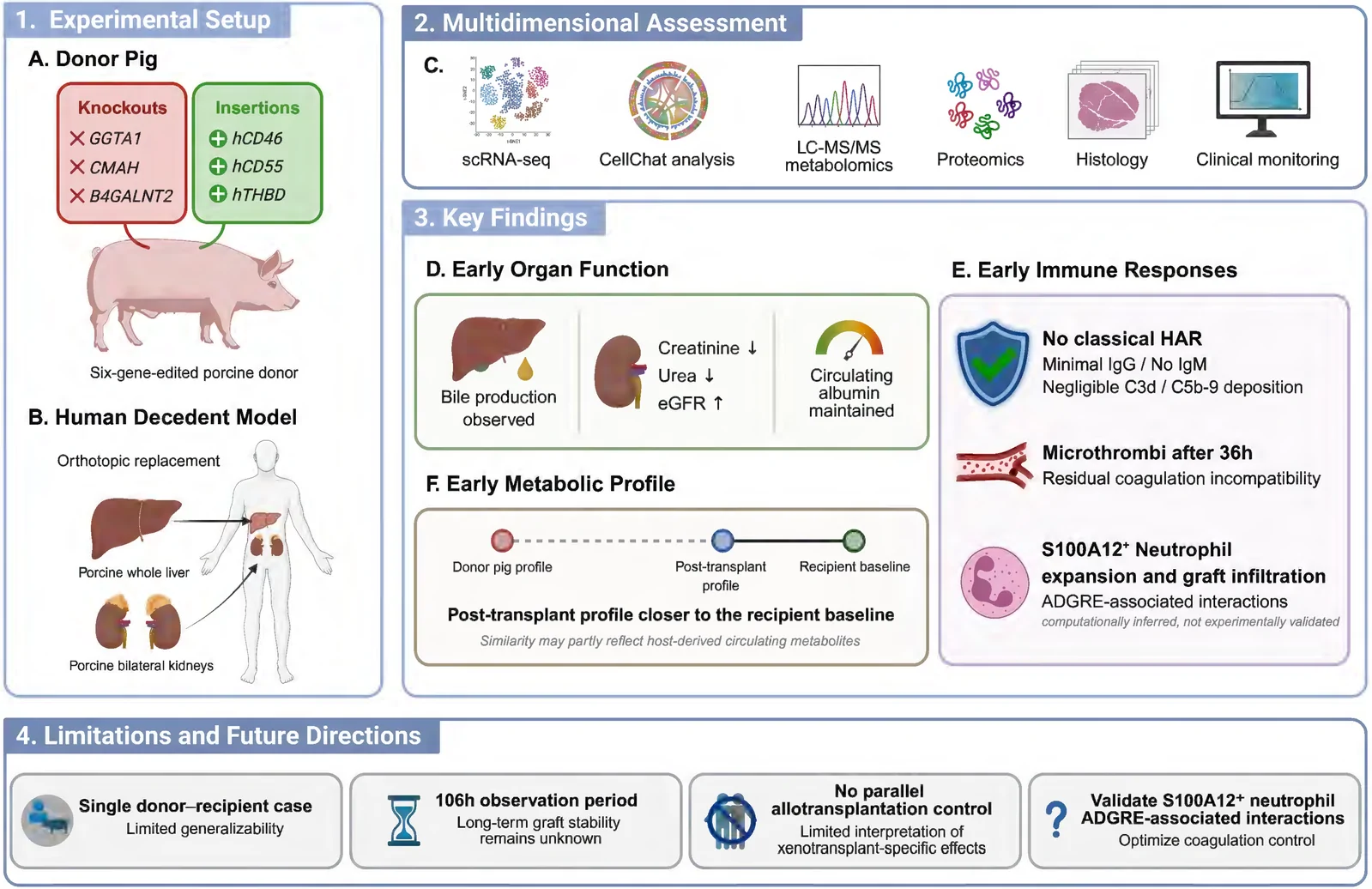
** Orthotopic multi-organ xenotransplantation involving a porcine whole liver and bilateral kidneys in a human decedent model. (A)** A six-gene-edited donor pig carrying knockouts of *GGTA1*, *CMAH*, and *B4GALNT2* together with insertion of *hCD46*, *hCD55*, and *hTHBD* was used for orthotopic multi-organ xenotransplantation. **(B)** In the human decedent model, the native liver and kidneys were replaced orthotopically with a porcine whole liver and bilateral kidneys.** (C)** Multidimensional assessment included scRNA-seq, CellChat analysis, LC-MS/MS metabolomics, proteomics, histology, and clinical monitoring. **(D)** Early functional evaluation demonstrated bile production, maintenance of circulating albumin levels, reduced serum creatinine and urea concentrations, and increased eGFR. **(E)** Classical HAR was not observed during the early post-transplant period, although microthrombi appeared after 36h, indicating residual coagulation incompatibility. Immune analyses identified expansion and graft infiltration of S100A12⁺ neutrophils and suggested ADGRE-associated interactions as a potential pathway involved in early innate immune responses. **(F)** Metabolomic profiling showed that post-transplant metabolic signatures remained more closely aligned with the recipient baseline than with the donor pig profile. The study was limited by its single donor-recipient design, a 106h observation period, and the absence of a parallel allotransplantation control. Future studies should validate the role of S100A12⁺ neutrophils and ADGRE-associated interactions and establish strategies for improving coagulation compatibility. Abbreviations: ADGRE: adhesion G protein-coupled receptor E family; C3d: complement component 3d; C5b-9: terminal complement complex; eGFR: estimated glomerular filtration rate; HAR: hyperacute rejection; IgG: immunoglobulin G; IgM: immunoglobulin M; LC-MS/MS: liquid chromatography-tandem mass spectrometry; scRNA-seq: single-cell RNA sequencing.
